# Case report: Monoclonal CGRP-antibody treatment in a migraine patient with a mutation in the mitochondrial single-strand binding protein (SSBP1)

**DOI:** 10.3389/fneur.2022.958463

**Published:** 2022-09-15

**Authors:** Katharina Kaltseis, Elisabetta Indelicato, Gregor Broessner, Sylvia Boesch

**Affiliations:** Department of Neurology, Medical University of Innsbruck, Innsbruck, Austria

**Keywords:** headache, migraine, mitochondrial disease, prophylactic treatment, CGRP, case report

## Abstract

**Background:**

There is a growing body of mitochondrial disorders that are associated with headaches, albeit only one of them is currently listed in the latest International Classification of Headache Disorders, 3rd edition (ICHD-3). Headache frequency and headache presentation can vary widely in this respective patient group. Acute and preventive migraine treatment can be quite challenging—the use of several established medications is often limited due to their side effects in the setting of mitochondrial dysfunction and multi-organ disease.

**Case presentation:**

Along with a review of the literature on treatment options in patients with mitochondrial disorders and migraine headaches, we present the case of a 23-year-old male with a homozygous mutation in the mitochondrial single-strand binding protein (*SSBP1*) with chronic migraine with aura. After failing several standard of care prophylactics due to either side effects or inefficacy, he was successfully treated with a monoclonal anti-CGRP-antibody as a preventive migraine treatment. The monoclonal antibody was well tolerated and showed adequate efficacy with a sustained > 50% reduction in monthly headache days after 3 years of treatment.

**Conclusion:**

Migraine is often challenging to treat in patients with mitochondriopathy due to therapy-limiting comorbidities. Monoclonal CGRP-antibodies might be a safe treatment option in the prevention of migraine headaches in patients with a mitochondrial disorder.

## Introduction

Mitochondrial disorders are characterized by defects in oxidative phosphorylation and caused by mutations in genes in the nuclear DNA (nDNA) and mitochondrial DNA (mtDNA). Mitochondriopathies can present with multisystemic manifestations including non-neurological symptoms with cardiac, renal, muscle, and digestive involvement as well as neurological symptoms, such as epileptic seizures, ataxia, and headache (1).

The presence of migraine with and without aura and other headache disorders, such as tension-type headaches, is well described in mitochondrial diseases, such as chronic progressive external ophthalmoplegia (CPEO), myoclonic epilepsy with ragged-red fibers (MERRF) and mitochondrial encephalomyopathy, lactic acidosis, and stroke-like episodes (MELAS) syndrome (2–5). Headache prevalence in this patient group seems to be higher than in the general population (3). In MELAS, between 50 and 90% of the patients report headaches (3, 5, 6), however, headache frequency and headache presentation can vary widely in this respective patient population. Although headache is associated with several mitochondriopathies, only one of them is currently listed in the latest International Classification of Headache Disorders, 3rd edition (ICHD-3) (7). Headaches associated with MELAS are categorized as secondary headaches attributed to genetic vasculopathy and can be either present as migraine attacks with or without aura or headaches in connection with seizures or/and neurological deficits (7).

Compared with other migraineurs, patients with mitochondriopathies often report inadequate response to acute medication (6). Moreover, the use of several established medications for both prevention and acute treatment of migraine is limited due to their side effects in the setting of mitochondrial dysfunction and multi-organ disease. Therefore, due to the lack of scientific evidence and data, it is challenging to offer these patients an effective and well-tolerated therapy for their headaches.

Herein we describe the case of an adult patient with a mitochondrial disorder, who was successfully treated with a calcitonin gene-related peptide (CGRP)-antibody for the prevention of migraine attacks. Written informed consent for the publication was given by the patient. The present patient was reported for the first time in the article from Del Dotto et al., though without a detailed clinical description of symptoms and response to therapy.

Furthermore, we reviewed the literature for treatment options for migraine headaches in patients with mitochondriopathies.

## Case presentation

We present a young male who was first referred to our outpatient clinic for rare movement disorders in 2009 at the age of 23. Since early childhood, the patient has had sensorineural hearing loss and retinitis pigmentosa, ultimately leading to severe visual impairment and deafness. Stance and gait ataxia as well as dyspraxia were present since the age of 4, however without progression over the years. Since the age of 6, the patient suffered from recurrent headache attacks, featuring a pulsating quality, unilateral presentation, and severe pain intensity. Headaches were accompanied by photophobia, nausea, as well as vomiting and were preceded by visual aura symptoms—hence, fulfilling the ICHD-3 diagnostic criteria for episodic migraine with aura (7).

**Figure 1:**
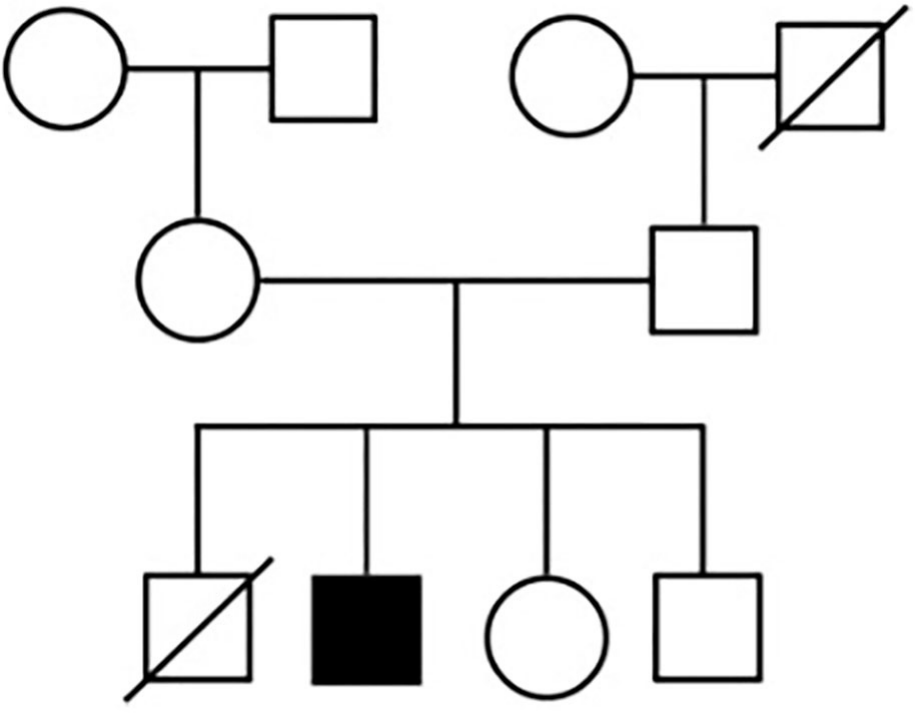
Pedigree of the patient's family.

The hypertrophic cardiomyopathy (New York Heart Association Class II), for which the patient received treatment with nebivolol 1.25 mg/day, was diagnosed in childhood. Renal impairment (creatinine 1.77 mg/dl), as well as scoliosis, were present from an early age and progressive over the years. Despite the manifold debilitating symptoms, such as mild cognitive impairment, the patient was able to cope with activities of daily living. The patient's family history is illustrated in Figure 1. It was unremarkable on the maternal and paternal sides, however, one brother died 11 days after birth (cause unknown).

At the age of 13, a muscle biopsy of the quadriceps revealed cytochrome-oxidase negative fibers but no ragged-red-fibers. Biochemical evaluation detected the reduced activity of complex I and complex III.

**Figure 2:**
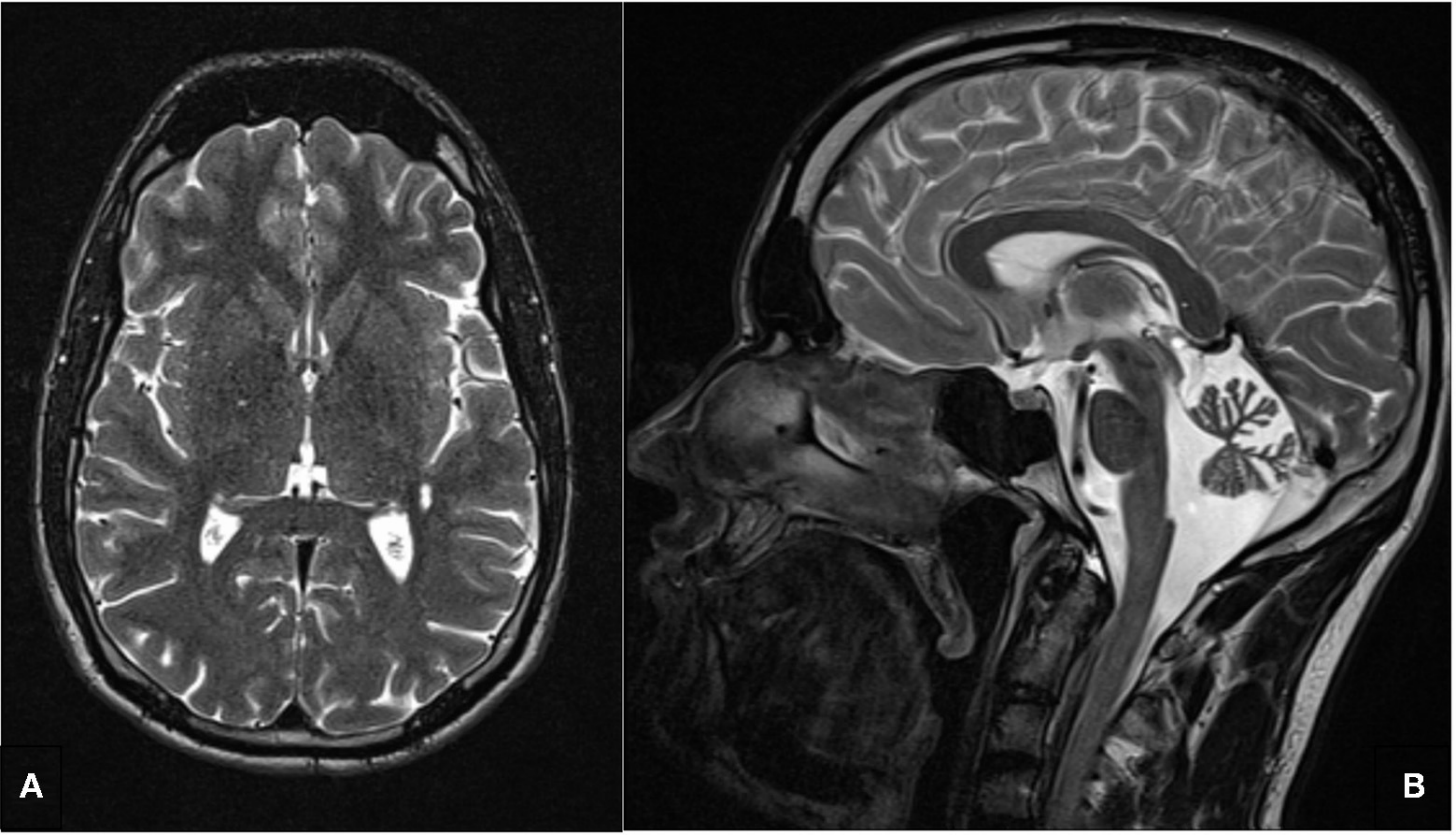
T2 weighted cerebral magnetic resonance image. **(A)** No intraparenchymal signal alterations in the axial image. **(B)** Cerebellar atrophy, particularly of the vermis cerebelli.

Repeated cerebral magnetic resonance imaging (cMRIs) revealed cerebellar atrophy, particularly of the vermis cerebelli (Figure 2) without intraparenchymal signal alterations. Magnetic resonance spectroscopy (MRS) displayed normal spectra and no lactate peak in supratentorial brain regions, suggesting no anaerobic metabolism and therefore no circulatory disorders. This finding is in line with the absence of stroke or stroke-like-episodes in the patient. Electroencephalography (EEG) showed diffuse intermittent slowing without epileptiform discharges.

Based on the clinical and diagnostic findings, a mitochondrial disease was suspected. Initial genetic testing was negative for Friedreich's-Ataxia, Fabry's disease, and the most common MELAS mutation, *3243 A*>*G*. Then, a whole exome sequencing (WES) was performed and a homozygous mutation in the mitochondrial single-strand binding protein (*SSBP1*), *c.394A*>*G* (p.I132V) was identified. A detailed description of the spectrum and the functional impact of *SSBP1* mutations is demonstrated in the study by Del Dotto et al. (8). The patient presented here is the index patient in family 5 in this respective article.

### Migraine and therapeutic approach

In 2012, frequent migraine attacks (ranging from 2 to 3 days/week) led to an enormous impairment of the patient's quality of life. The patient's stance and gait ataxia aggravated during migraine attacks, and he suffered from a prolonged postdromal phase, where he experienced severe fatigue and a decline in cognitive functions. Due to renal insufficiency, options for acute treatment were limited. The patient used frovatriptan 2.5 mg and metamizole 500–1,000 mg, both with poor effect. As prophylactic therapy, flunarizine 10 mg/day was started with sufficient treatment response and tolerability, resulting in a significant reduction of monthly migraine days. However, after 3 years, the headache frequency increased, despite regular prophylaxis intake, indicating the loss of treatment response. Flunarizine was withdrawn and, after consulting a cardiologist, the premedication with nebivolol was changed to bisoprolol 2.5 mg, as better evidence on its efficacy in migraine prevention has been reported (9, 10). Due to low blood pressure, increasing the dosage of bisoprolol was not attempted, yet the number of migraine days declined satisfactorily.

In 2019, the patient experienced another exacerbation of the monthly migraine days—more than 20 days/month were reported—fulfilling the diagnostic criteria for chronic migraine, despite ongoing therapy with bisoprolol. Therefore, an additional prophylactic treatment with galcanezumab 120 mg monthly (with a 240 mg loading dose) was started. After 2 months of treatment, the patient reported a >50% reduction in the monthly migraine days. Thereafter, he even experienced headache-free months. No side effects were reported. During the follow-up till 2022, a sustained satisfactory effect of galcanezumab was observed, without side effects on renal and cardiac function.

## Literature review

We performed a literature search of the electronic database PubMed on treatment options for patients with mitochondrial disorders and experiencing migraine headache. We used the search terms “mitochondrial disorder,” “mitochondriopathy/mitochondriopathies,” and “migraine/headache,” and “treatment/therapy” and included results from January 1985 until February 2022. The language was restricted to English and full text only. No randomized controlled trials are available—current treatment options are based on case reports and small case series. Altogether, we identified 4 other cases, comprising a total of 65 patients, on the preventive and acute treatment of migraine headaches in patients with a mitochondrial disorder (as shown in Table 1). The male to female ratio was 1:2.8. Phenotypes included MELAS, CPEO, MERRF, Leigh Syndrome, and other mitochondrial diseases. Information on the presentation of migraine (headache characteristics and accompanying symptoms) was not fully reported in the studies. Similarly, there was insufficient information about comorbidities, such as stroke-like-episodes (SLE) or epilepsy. Only two studies reported the use of prophylactic medications. In the study by Tiehuis et al., prophylactic migraine treatment (propranolol and metoprolol) was used by four patients, and which was partially effective for two of them (6). In the case report by Naegel et al., the patient used several preventive drugs. Two (topiramate and onabotulinumtoxinA) showed no efficacy and flunarizine had to be discontinued due to side effects. Regarding acute treatment, various drugs were used by the patients, among them triptans, which seemed to be well tolerated. The efficacy of acute medication was often not adequate—some patients had to use triple medication, which was still not effective (6).

**Table 1:** An overview of the reports on the use of acute or prophylactic migraine treatment in patients with a mitochondrial disorder and their clinical symptoms.

**Source**	**No. of cases**	**% Female**	**Genotype**	**Phenotype**	**MHD**	**A**	**UL**	**P**	**PTP**	**PNP**	**N**	**V**	**DG**	**PT**	**AT**	**abnormal EEG**	**Comorbidities**
Present case	1	0	c.394A > G	Other MD	10–12	+	+	+	+	–	+	+	EM	Flunarizine, bisoprolol, galcanezumab	Analgesics, Triptan	+	Cardiomyopathy, Retinitis pigmentosa, renal impairment, stand and gait ataxia
Naegel et al. (11)	1	100	m.3243A > G	MELAS	10–20	–	+	+	+	+	+	+	CM	Topiramate, onabotulinumtoxin, flunarizine, erenumab	Triptans	+	SLE
Tiehuis et al. (6)	29	83	m.3243A > G and non - m.3243A > G	MERRF; MELAS; Leigh Syndrome;	n.a.	12	n.a.	12	23	18	20	8	n.a.	Metoprolol, propranolol	Triptan, NSAIDs, Acetaminophen, combinational analgesics	n.a.	Diabetes, Impaired hearing, impaired vision, GIT problems, muscle related problems
Vollono et al. (4)	33	64	m.3243A > G m. 8344A > G m. 8356T > C single/ multiple mtDNA deletion OLGI; TYMP m.9242insA	MELAS; CPEO; MERRF; Other MD; MNGIE	3.9 ± 6.3	6	22	n.a.	23	22	16	6	EM	n.a.	NSAIDs; Acetaminophen; Triptan, Codeine; Analgesic	20	SLE; epilepsy; myoclonus; stroke
Iizuka et al. (12)	2	100	n.a.	MELAS	n.a.	–	1	+	1	–	+	+	EM	n.a.	Triptan	1	SLE

A, Aura; AT, acute treatment; CM, chronic migraine; CPEO, Chronic progressive external ophthalmoplegia; DG, Diagnosis; EEG, electroencephalogram; EM, episodic migraine; GIT, gastrointestinal; MD, mitochondrial disease; MELAS, mitochondrial encephalomyopathy, lactic acidosis, and stroke-like episodes; MERRF, myoclonic epilepsy with ragged-red fibers; MHD, mean headache days/month; MNGIE, mitochondrial neurogastrointestinal encephalomyopathy; N, nausea; n.a., not applicable; NSAIDs, non-steroidal anti-inflammatory drugs; PT, prophylactic treatment; SLE, stroke-like-episodes; UL, unilateral headache; PTP., photophobia; PNP, phonophobia; V, vomiting.

## Discussion

We present a patient with a genetically confirmed mitochondrial disorder [homozygous missense change in *SSBP1; c.394A*>*G* (p.I132V)] with episodic/chronic migraine with aura who received galcanezumab for preventive migraine treatment, after having failed two standard-of-care treatment approaches. Herein, we describe the second case of the successful and safe use of a monoclonal anti-CGRP-antibody in a patient with a mitochondrial disease (11), and we report for the first time a long-term follow-up of 3 years. Treatment with galcanezumab was well tolerated and showed adequate efficacy with a sustained > 50% reduction in monthly headache days.

Due to the possible involvement of various organ systems, especially those with high energy requirements, several medications need to be used with caution in this patient population (13). First-line medications for acute migraine treatment, such as non-steroidal anti-inflammatory drugs (14), are contraindicated in the setting of renal impairment, as it was the case in our patient. The frequent use of acetaminophen as acute medication may deplete glutathione and cause hepatopathy (13). Triptans seem to be effective and well tolerated in such patients and therefore might be considered for the acute treatment of migraine attacks (11, 12).

For migraine prevention, antiepileptic drugs (AEDs) have been proven beneficial (15). However, some AEDs, such as valproic acid are mitochondrion-toxic and could, moreover, lead to irreversible liver failure (13, 16). Topiramate as prophylaxis could cause lactate acidosis and therefore has also to be used with reluctance. Coenzyme Q_10_, magnesium, and riboflavin (vitamin B_2_) are substances that have shown only minor benefits in reducing headache intensity and headache duration in migraine patients (17–20). Since all of them are also used in the treatment of mitochondrial disorders (21), a common underlying pathogenic mechanism could be suspected—not only due to similarities in therapeutic approaches but also based on genetic and biochemical evidence (22). Up to date, reports on the effectiveness of these agents on migraine in patients with mitochondriopathies are lacking.

Mitochondrial dysfunction is considered to increase neuronal excitability, thus lowering the threshold for a migraine attack (23). Imaging studies showed impaired mitochondrial oxidative phosphorylation during and between migraine attacks, implying a disrupted energy metabolism in migraineurs (24). The dysfunction of mitochondria is linked to an increase in reactive oxygen species (ROS). In addition, in migraine patients, the antioxidative capacity is lowered, exposing them more easily to higher levels of oxidative stress (23). Many potential migraine triggers, such as fasting, hypoxia, and sleep deprivation, enhance oxidative stress. CGRP, a neuropeptide that seems to play an important role in the development of migraine, is assumed to be released as a response to oxidative stress (25, 26) and is responsible, among other transmitters, for the pain signaling in the trigeminovascular system (27). Blocking CGRP or its receptor with monoclonal antibodies or gepants aborts migraine attacks and reduces their frequency (28). As CGRP is a potent vasodilator and supposedly has cardioprotective effects, an increased risk for cardiovascular events could be feared when inhibiting the CGRP pathway (29). This would also be of relevance in patients with mitochondriopathies, as cardiac involvement is often the predominant clinical manifestation. However, recent data showed a safe cardiovascular profile for CGRP-receptor and CGRP-ligand blocking monoclonal antibodies, respectively (30–32). Regular follow-up over 3 years of therapy with galcanezumab reported no worsening of cardiac functions or cardiac symptoms in our patient.

Headaches account for one of the five most debilitating symptoms of mitochondrial diseases (6). Headaches, especially migraine, have a substantial impact on social functioning and everyday life in patients with mitochondriopathies (5). However, both, acute as well as a preventive treatment for migraine in patients with mitochondrial disease are challenging—data on the use and efficacy of medication are scarce. The literature search revealed only 4 other reports on the use of preventive and/or acute migraine treatment in this respective patient group, and these were either ineffective or had to be discontinued due to side effects. Frequently, it is not possible to use the medication in the appropriate dosage due to therapy-limiting comorbidities. Due to the lack of randomized control trials and particularly due to the heterogenous presentation of mitochondrial disorders, treatment recommendations cannot be provided, and therapy remains empirical (33).

The strength of the study presented here is the detailed description of the patient's migraine headache, comorbidities, imaging and genetic findings, the rationale of the used acute and preventive treatments, as well as the documentation of the long-term therapeutic outcome. The efficacy, tolerability, and safety of a monoclonal CGRP-antibody in the prevention of migraine headaches could be demonstrated in our patient with a homozygous missense change in *SSBP1*.

However, further research, including randomized controlled trials and a precise and comprehensive patient as well as headache characterization is needed to provide treatment guidelines for patients with migraine due to mitochondrial disorders.

## Data availability statement

The original contributions presented in the study are included in the article, further inquiries can be directed to the corresponding author.

## Ethics statement

The studies involving human participants were reviewed and approved by the Ethics Committee of the Medical University of Innsbruck. The patients/participants provided their written informed consent to participate in this study. Written informed consent was obtained from the individual(s) for the publication of any potentially identifiable images or data included in this article.

## Author contributions

KK: conceptualization and writing—original draft. EI and GB: writing—review and editing. SB: conceptualization, supervision, and writing—review and editing. All authors contributed to the article and approved the submitted version.

## Conflict of interest

The authors declare that the research was conducted in the absence of any commercial or financial relationships that could be construed as a potential conflict of interest.

## Publisher's note

All claims expressed in this article are solely those of the authors and do not necessarily represent those of their affiliated organizations, or those of the publisher, the editors and the reviewers. Any product that may be evaluated in this article, or claim that may be made by its manufacturer, is not guaranteed or endorsed by the publisher.
